# *Elongating, entwining, and dragging*: mechanism for adaptive locomotion of tubificine worm blobs in a confined environment

**DOI:** 10.3389/fnbot.2023.1207374

**Published:** 2023-08-29

**Authors:** Taishi Mikami, Daiki Wakita, Ryo Kobayashi, Akio Ishiguro, Takeshi Kano

**Affiliations:** ^1^Research Institute of Electrical Communication, Tohoku University, Sendai, Japan; ^2^Graduate School of Engineering, Tohoku University, Sendai, Japan; ^3^Program of Mathematical and Life Sciences, Graduate School of Integrated Sciences for Life, Hiroshima University, Higashihiroshima, Japan

**Keywords:** swarm, entangled blob, active matter, soft robotics, adaptive locomotion

## Abstract

Worms often aggregate through physical connections and exhibit remarkable functions such as efficient migration, survival under environmental changes, and defense against predators. In particular, entangled blobs demonstrate versatile behaviors for their survival; they form spherical blobs and migrate collectively by flexibly changing their shape in response to the environment. In contrast to previous studies on the collective behavior of worm blobs that focused on locomotion in a flat environment, we investigated the mechanisms underlying their adaptive motion in confined environments, focusing on tubificine worm collectives. We first performed several behavioral experiments to observe the aggregation process, collective response to aversive stimuli, the motion of a few worms, and blob motion in confined spaces with and without pegs. We found the blob deformed and passed through a narrow passage using environmental heterogeneities. Based on these behavioral findings, we constructed a simple two-dimensional agent-based model wherein the flexible body of a worm was described as a cross-shaped agent that could deform, rotate, and translate. The simulations demonstrated that the behavioral findings were well-reproduced. Our findings aid in understanding how physical interactions contribute to generating adaptive collective behaviors in real-world environments as well as in designing novel swarm robotic systems consisting of soft agents.

## 1. Introduction

In nature, biological organisms (e.g., insects, fish, and birds) often swarm to improve their odds of survival, and the swarm behaves as if it were an individual system (Kennedy, 2006; Miura et al., 2022). These features have attracted the attention of researchers from various fields. For example, biologists have investigated the swarming behaviors at various scales of living systems, from bacteria (Verstraeten et al., 2008) to mammals (Lien et al., 2004). Active matter physics studies have investigated the collective dynamics of self-propelling particles such as camphor disks (Suematsu and Nakata, 2018). In the engineering field, swarm robotic systems made of multiple rigid robots from the micrometer scale (Hauert and Bhatia, 2014) to tens of centimeters (Brambilla et al., 2013), have been studied for engineering applications such as collective transport and pattern formation (Brambilla et al., 2013).

A swarm uses various types of local inter-individual interactions to perform ordered collective behaviors. For example, chemical cues are used for inter-individual interactions in ant trails (Wilson, 1962), whereas fluid dynamics are used for interactions in schools of fish and flocks of birds (Partridge and Pitcher, 1979; Bajec and Heppner, 2009). Physical connections between individuals are an intriguing type of interaction because they lead to the generation of nontrivial, versatile macroscopic patterns, and functions under various circumstances that have been studied in biology and active matter physics (Shishkov and Peleg, 2022). Well-known examples include fire ant rafts (Mlot et al., 2011), army ant bridges (Garnier et al., 2013), honeybee clusters (Peters et al., 2022), and larval aggregations (Sutou et al., 2011). In these systems, macroscopic structures suitable for survival are formed through physical connections that exploit the mechanical properties of individuals, enabling them to deform and move flexibly under environmental changes. Understanding the mechanisms that underlie physical-connection-based swarms is a popular research topic (Shishkov and Peleg, 2022).

Among physical-connection-based swarms, worm-shaped organisms form tight entanglements that coordinately perform adaptive locomotory patterns. As a slender and flexible individual (“worm”) has an enormous number of bodily degrees of freedom, its collective (“worm blob”) takes a variety of forms.

For example, *Caenorhabditis elegans* aggregates under certain conditions (Chen and Ferrell Jr, 2021), forms dynamic network structures (Sugi et al., 2019; Demir et al., 2020), and moves within aggregations (Demir et al., 2020). Caterpillars migrate collectively in a procession (Maronna et al., 2008; Sutou et al., 2011; Uemura et al., 2020). Blackworms (*Lumbriculus variegatus*), which are worm-shaped organisms with around 1 mm diameter, form yarn ball-like blobs by entangling with each other, migrating, and deforming into a collective (Ozkan-Aydin et al., 2021).

The mechanisms underlying the collective behavior of worm-shaped organisms have been discussed previously. Sugi et al. (2019) investigated the dynamic network formation in *C. elegans* using behavioral experiments and mathematical modeling. Deblais et al. (2020) investigated the mechanism of phase separation of *Tubifex tubifex* experimentally as well as theoretically. Ozkan-Aydin et al. (2021) successfully reproduced the temperature-dependent behavior of blackworm blobs using a simple robotic model. Furthermore, Nguyen et al. (2021) built a theoretical model of the thermotaxis of blobs and explained how the behavior changed depending on the parameters through simulations. However, previous studies on the locomotion mechanisms of swarms of worm-shaped organisms have focused on locomotion in flat environments, and it remains unclear how they move in real-world environments, which have confined spaces and convex and concave environments, by exploiting interindividual physical interactions.

In the present study, we investigated the mechanism of the adaptive locomotor behavior of worm-shaped organisms in a confined environment. We focused on a group of tubificine worms that exhibit a wide range of behaviors similar to those of blackworms (Ozkan-Aydin et al., 2021). Behavioral experiments were conducted to study adaptive locomotion in artificially created confined environments. Subsequently, based on the behavioral findings, we built a simple two-dimensional (2D) agent-based model and performed simulations to validate it. Our findings can also lead to the development of a novel swarm robotic system consisting of multiple soft and deformable agents.

The remainder of this paper is organized as follows. In Section 2, we investigate blob behavior in a flat environment (Section 2.1) and a confined environment (Section 2.2). Section 2.3 presents the locomotion patterns of one or a few worms on a flat plane to understand the locomotory mechanism. Section 2.4 presents our working hypothesis for their adaptive behavior in a confined environment. In Section 3, we propose a mathematical model that captures the essence of the blob behavior. In Section 4, we demonstrate through simulations that our model can recapitulate behavioral findings with common parameter values. Finally, we discuss the future perspectives and limitations of our model (Section 5).

## 2. Biological experiments

Tubificine worms (Annelida: Tubificinae) were obtained from Aquarium Time (Miyagi, Japan) and Aqua Field (Tokyo, Japan). Through anatomical classification, we confirmed that this worm colony mainly consisted of *Limnodrilus hoffmeisteri* Claparède, 1862, *Limnodrilus udekemianus* Claparède, 1862, and *Tubificine* sp. The worms were kept in a box (350 × 200 × 120 mm) filled with circulating water (height≥200 mm) at 20°C for less than two weeks prior to the experiments. Water was changed daily.

As preliminary experiments (Section 2.1), we qualitatively observed in a flat environment whether tubificine worms gathered into blobs similarly to *T. tubifex* (Deblais et al., 2020; Section 2.1.1) and whether the blob escaped from an aversive stimulus as in blackworms (Ozkan-Aydin et al., 2021; Section 2.1.2). Then, we performed the main experiments in this study; we investigated how a blob moves in confined environments (Section 2.2). Finally, we observed the locomotory patterns of one or more worms to understand their locomotion mechanisms (Section 2.3).

The experimental arena was set on a flat table that was illuminated from above. Unless otherwise specified, the experiments were recorded using video cameras (GZ-F270-W, JVC, Japan).

### 2.1. Preliminary experiments: collective behavior of tubificine worm blobs in a flat environment

#### 2.1.1. Blob formation

The worms aggregated to form blobs in a flat environment. An acrylic box (390 × 290 mm; As One, Japan) filled with water (226 ml, 2 mm in height) and scattered worms (60 *g*) were used.

**Figure 1:**
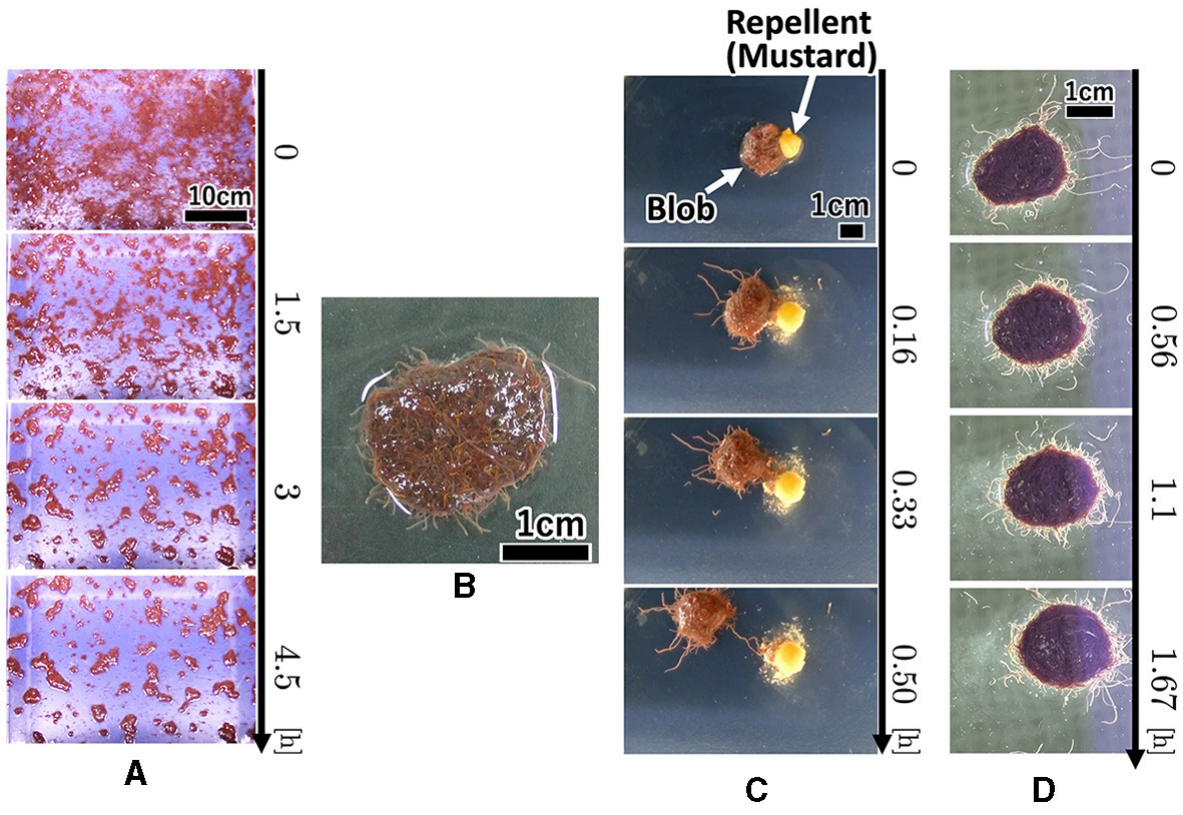
Collective behaviors of the tubificine worms' blob on flat planes. **(A)** Blob formation. **(B)** Formed blob (1*g*). **(C)** Collective escape from the repellent [Japanese mustard (S & B Shokuhin Co. Ltd., Japan)].

Each worm initially moved randomly; however, once it contacted nearby worms, they became entangled with one another (Figure 1A, 0–90 min; Supplementary Video 1). Subsequently, many small blobs formed (90 min). These blobs gradually increased in size by merging with the surrounding worms and blobs (90–270 min). The number of blobs gradually decreased. Worms in a blob did not move actively; however, worms at the edge of the blob wiggled their tails outside the blob, with their heads inside the blob (Figure 1B; Supplementary Video 2).

#### 2.1.2. Collective escape from aversive stimulus

We investigated whether the blobs exhibited a collective escape from aversive stimuli. We used a petri dish with 150 mm diameter filled with water (10 ml, 0.57 mm height). We placed a blob (1*g*) at the center and added Japanese mustard (0.3*g*; S & B Shokuhin Co. Ltd., Japan; hereafter referred to as mustard) at the edge of the blob as an aversive stimulus.

The surface of the blob fluctuated significantly when mustard was added (Figure 1C, 0–10 min; Supplementary Video 3). Some worms removed their heads from the blob and moved to the opposite side of the mustard (10 min). Subsequently, the blob escaped the stimulus (20 min). It escaped straight from the mustard while maintaining its hemispherical shape (20–30 min).

### 2.2. Collective self-transport by exploiting pegs in a confined environment

**Figure 2:**
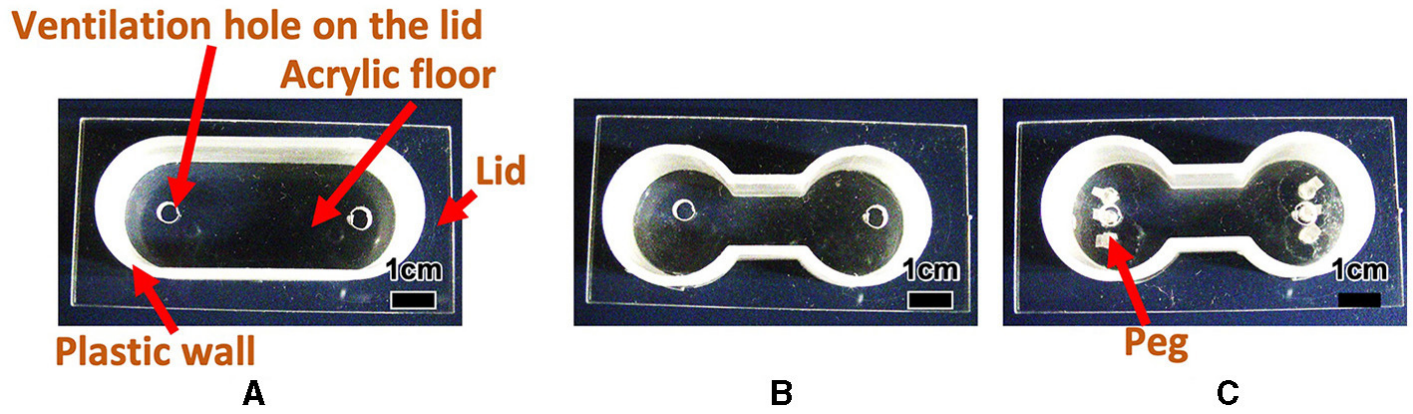
Experimental cases used in Section 2.2. **(A)** Oval shape case. **(B)** Dumbbell-shaped case without peg. **(C)** Dumbbell-shaped case with pegs.

Tubificine worms live in complex natural environments, including confined spaces and convex and concave environments. Although tubificine worms move while maintaining their hemispherical shape in a flat environment, as discussed in Section 2.1.2, this may not be the case in such complex environments. Thus, we performed behavioral experiments in environments with various boundary conditions to understand how they move in complex environments. Specifically, to highlight behavioral tendencies and make the analysis, the following three conditions were examined: (i) an oval-shaped case with no pegs (Figure 2A), (ii) a dumbbell-shaped case with no peg (Figure 2B), and (iii) a dumbbell-shaped case with several pegs (Figure 2C). Our expectation is that the one-way trips for case (ii) are smaller than those for case (i) because the narrow path hinders the blob from moving, whereas the one-way trips for case (iii) are larger than those for case (ii) because the worms can utilize the pegs to move. Sections 2.2.1 and 2.2.2 compare cases (i) and (ii) and cases (ii) and (iii), respectively.

#### 2.2.1. Blob movements in oval- and dumbbell-shaped cases

We made oval (Figure 2A; Major axis 50 mm, minor axis 20 mm, height 15.5 mm), and dumbbell-shaped cases (Figure 2B; Room: Inside radius 10 mm, height 15.5 mm; Distance between the centers of the two rooms 30 mm; Narrow aisle: Inside width 10 mm, height 15.5 mm), and compared the behaviors of blobs between the two cases. We added water 6.39 ml (7 mm height) to the oval and 5.16 ml (7 mm height) to the dumbbell-shaped cases. Subsequently, we added a blob of 1 *g*, a diameter of ~15 mm to one side of each case. Here, we note that the diameter of the room in the dumbbell-shaped case was sufficiently wide to hold the 1 *g* blob; however, the width of the narrow aisle was so narrow that the blob could not pass through while maintaining its hemispherical shape. In the oval-shaped case, the room diameter is the same as that in the dumbbell-shaped case.

Thus, the blob had to deform so that it passed through the narrow aisle of the dumbbell-shaped case, whereas deformation was unnecessary in the oval case. An acrylic lid with ventilation holes was placed on each case to prevent evaporation. The subsequent progress of 10 trials was observed for 66 h using GoPro9 (time-lapse mode, 5 s, 4 K) for each condition. Blob behavior was evaluated quantitatively by counting the number of one-way trips.

**Figure 3:**
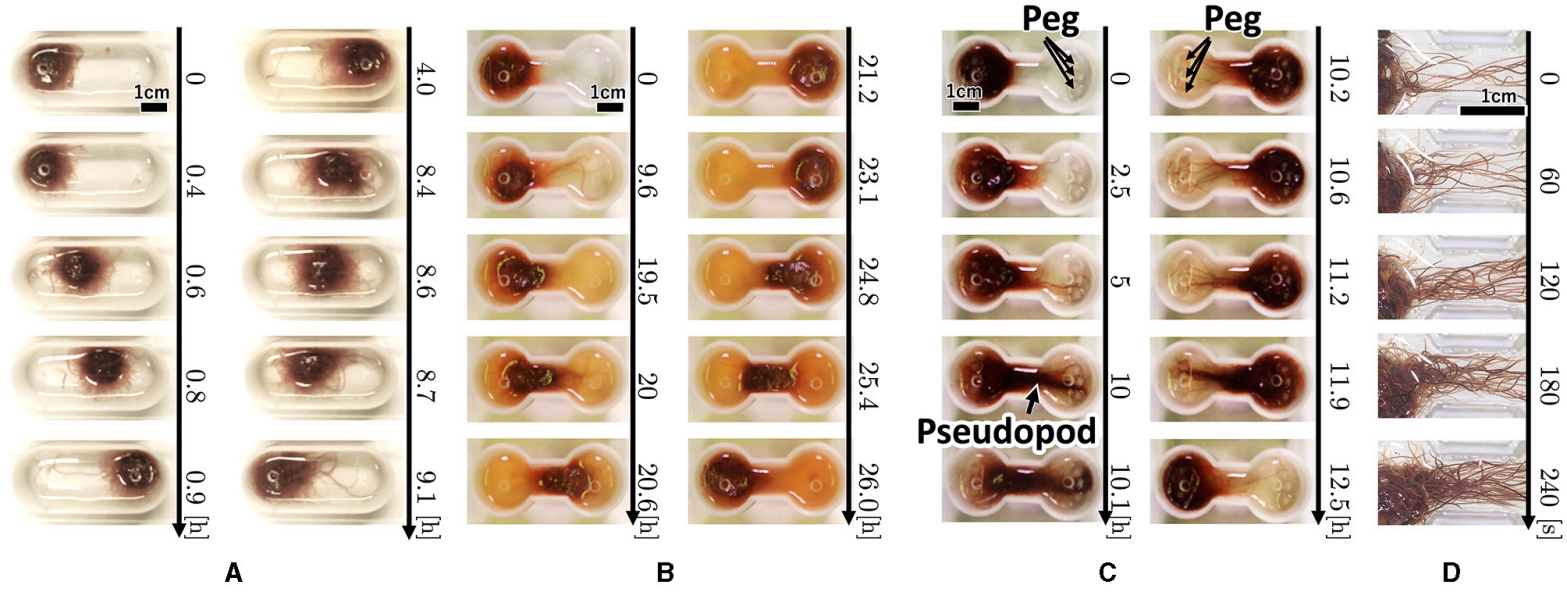
Snapshot of the collective locomotory behavior of the tubificine worms' blob in the confined environment. **(A)** Oval-shape case. **(B)** Dumbbell-shaped case without pegs. **(C)** Dumbbell-shaped case with pegs. **(D)** Snapshot focusing on the pseudopod formation.

**Figure 4:**
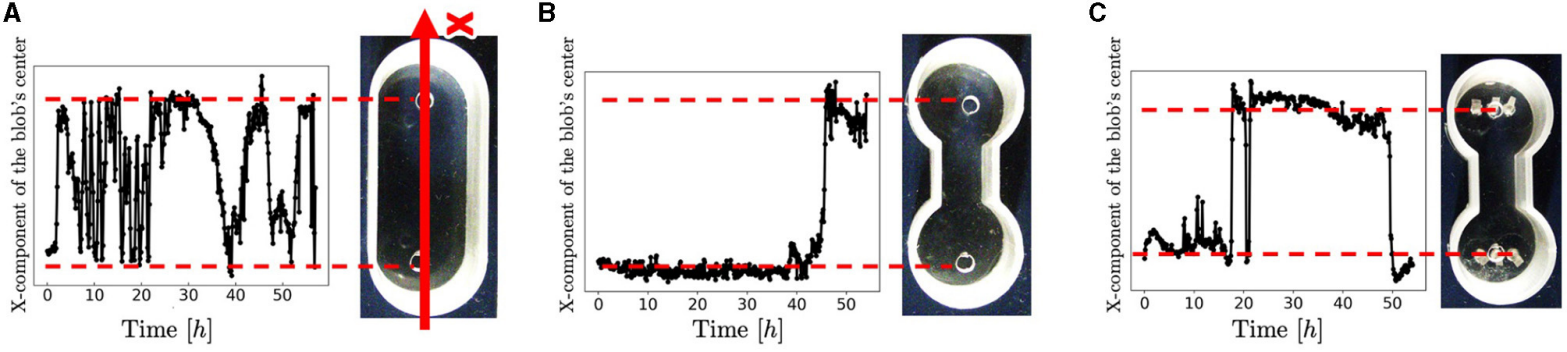
Trajectories of the blobs' travel. **(A)** Oval-shape case. **(B)** Dumbbell-shaped case without pegs. **(C)** Dumbbell-shaped case with pegs. These trials differ from those shown in Figure 3. We extracted the areas where blobs were present as pixels and calculated the center of mass. The code was implemented in Python 3.

Snapshots of a representative trial for each condition (Figure 3) are explained below. For the oval-shaped case (Figure 4A; Supplementary Video 4), the blob started to move after a time, even though we did not provide any stimuli (0–0.4 h). The blob moved straight to the other side (0.4–0.9 h). Subsequently, it returns to its initially placed straight side (4.0–9.1 h). It is unclear why blobs or worms did not remain in a certain location despite the absence of mustard. We speculated that these worms released a substance that functions as a repellent. The trajectory of the blob's center in an oval case trial (Figure 4A) showed an irregular oscillatory pattern. The blob frequently moved back and forth from 0 to 21 *h*; however, after 21 *h*, the motion became inactive.

**Figure 5:**
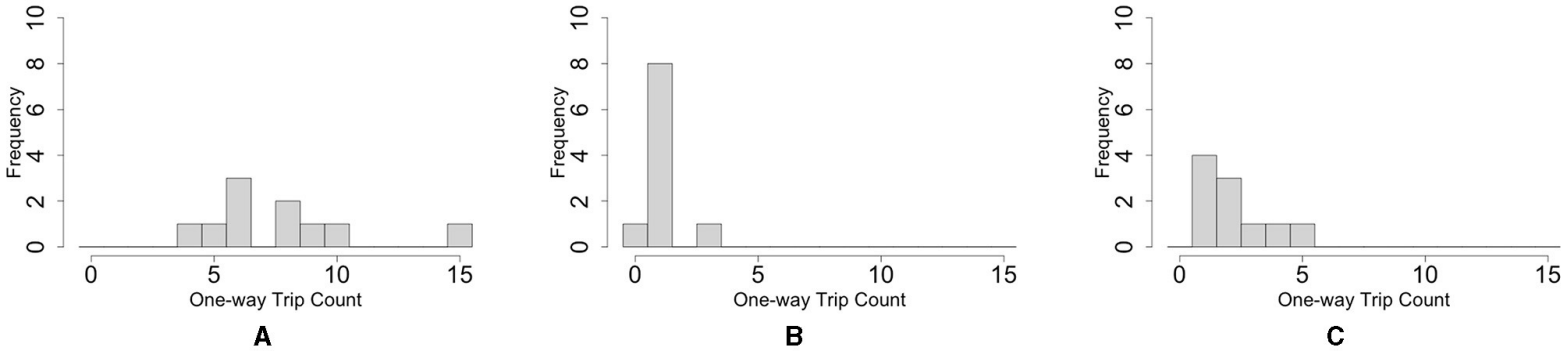
Histograms of the number of the blob one-way trips. **(A)** Oval-shape case. **(B)** Dumbbell-shaped case without pegs. **(C)** Dumbbell-shaped case with pegs. For each case condition, trips in 10 cases were counted within 66 h. These trials differ from those shown in Figure 3.

In the dumbbell-shaped case (Figure 3B; Supplementary Video 5), some worms got out of the blob (0–9.6 h). The blob could not pass through the narrow aisle for a certain amount of time (9.6–19.5 h). Subsequently, the entire blob was deformed to fit the shape of the case and moved to another room (19.5–21.2 h). After the blob reached the room, it returned to the initial room using a similar process (23.1–26.0 h). These results suggest that a hemispherical blob can deform and pass through an aisle; however, passing through the aisle by the blob took a long time. The trajectory of the blob's center in the dumbbell case trial (Figure 5B) showed that the blob had stayed in the initial side of the case from 0 to 40 *h* and then traveled only one-way (41–44 h).

The histogram of the number of one-way trips of the blobs shows that the maximum number in the oval-shaped case experiment was 15 trips, and the mode was six trips (Figure 5A; Supplementary Video 6). In contrast, the maximum number in the dumbbell-shaped case is three trips, and mode is on one trip (Figure 5B; Supplementary Video 7, bottom three rows). Statistical analysis showed that the number of one-way trips of the blobs in the dumbbell-shaped case was significantly lower than that in the oval case (*p* < 0.01, Mann–Whitney *U*-test).

#### 2.2.2. Comparison between with and without pegs

To examine whether worm blobs could adapt to a confined environment, we created a dumbbell-shaped case in which three pegs (1.5 × 1.5 × 10.0 mm) were anchored to each room (Figure 2C). The anchored pegs were so small that the worms could interact with them. We observed how the behaviors differed between with (Figure 2C) and without (Figure 2B) pegs.

Snapshots of representative dumbbell-shaped cases with anchored pegs (Figure 3C; Supplementary Video 8) show that several worms got out of the blob and expanded their bodies toward the opposite room in a similar manner as the condition of the dumbbell-shaped case without pegs (0–5 h). When several worms reached and entwined the pegs at their destination, other worms got out from the blob and joined a group of worms that had already reached the peg (5–10 h). This resulted in the formation of elongated worm bundles. Hereafter, we refer to this bundle as a “pseudopod.” The remaining blob was probably pulled by the pseudopod and deformed considerably, and the entire group was moved to another room (10.1–10.2 h). The blob was returned to the initial room using the same process (10.6–12.5 h). These results suggest that the blob can perform effective locomotion in a confined environment, such as a peg that is useful for locomotion.

Photographs (Figure 3D) and videos (Supplementary Video 9; taken with S9D, Leica, Germany; Eye lens: 1.0x, objective lens: 0.6x) with magnified view were taken to investigate the movement of the worm during the pseudopod formation in another trial wherein the dumbbell-shaped case with pegs (water: 2 ml, 2.75mm height) was used. Several worms first expand their bodies from the blob to a narrow path (0 s). During this process, the worms were aligned along the wall and occasionally entangled with each other, forming an immature pseudopod (0–60 s). Subsequently, other worms joined the pseudopod for growth. We observed that the elongated worms in the pseudopod were almost completely aligned, whereas those in the blob were oriented in random directions and entangled with one another. Over time, other worms were removed from the blob and joined the pseudopod (60–240 s). The trajectory of another representative trial of the dumbbell-shaped case with anchored pegs exhibited irregularities in the oscillatory period (Figure 4C). The blob first stayed on the initial side of the case from 0 to 17 *h*. Subsequently, one-way trips (17–21.5 h) were performed. Subsequently, it remained on one side for a while (21.5–47 h). Finally, a one-way trip is shown.

The histogram of the number of one-way trips of the blob in the case with pegs shows that the maximum number was five trips, and the mode was on one trip (Figure 5C; Supplementary Video 7, upper three rows). Statistical analysis showed that the number of one-way trips of the blobs in the case with pegs was significantly larger than that in the case without pegs (*p* < 0.05, Mann–Whitney *U*-test).

Summarizing Sections 2.2.1 and 2.2.2, worms tend to form hemispherical blobs in open spaces; thus, it is not feasible to pass them through an aisle with a width shorter than the diameter of the blob (Figure 5B). However, environmental heterogeneity (the existence of pegs) helped the blob deform and feasibly pass through the narrow aisle (Figure 5C).

### 2.3. Locomotory patterns of one or a few worms

To understand the mechanism of tubificine worm movement in more detail and to model locomotory behaviors, we investigated the locomotory patterns of one (Section 2.3.1) and several (Section 2.3.2) worms on a flat plane. We also observed the response of the worms to an aversive stimulus (see Section 2.3.3). We present a representative trial for each experiment because we acquired similar tendencies in several trials.

#### 2.3.1. Locomotion of a worm

**Figure 6:**
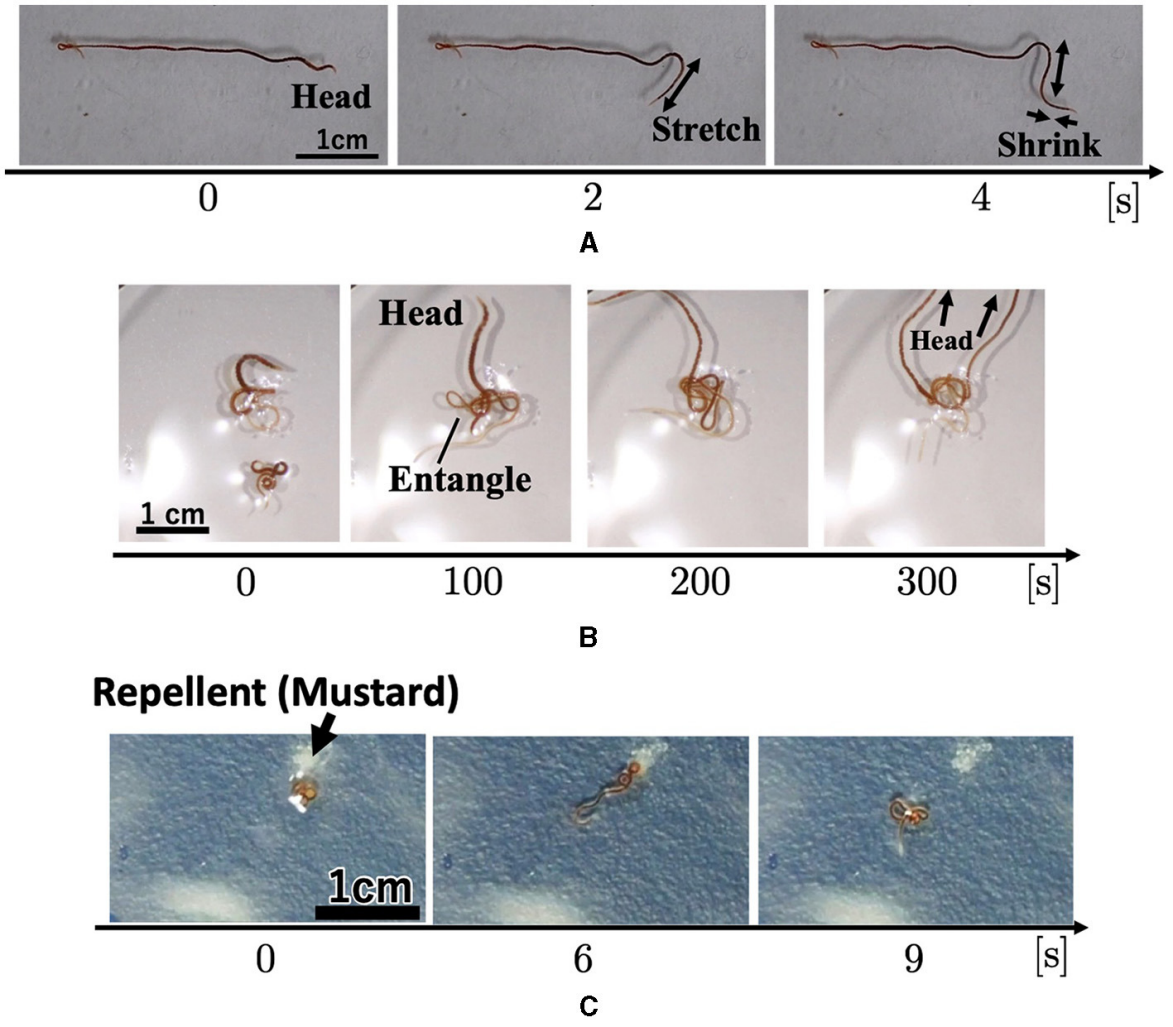
Locomotory patterns. **(A)** A worm locomotion. **(B)** Behavior of two worms. **(C)** Worm's response to the repellent mustard.

Worm locomotion was observed in a flat environment. The worm was placed in a 150 mm Petri dish filled with water (20 ml, 0.57 mm height). The worm propelled itself by propagating body waves of contraction and expansion from head to tail (Figure 6A; Supplementary Video 10) in a similar manner as earthworms (Gray and Lissmann, 1938) and blackworms (Ozkan-Aydin et al., 2021). In addition, we observed that the head turned frequently and randomly, similar to blackworms (Ozkan-Aydin et al., 2021).

#### 2.3.2. Locomotion of two worms

Two worms were placed in a 150 mm Petri dish filled with water (20 ml, 0.57 mm height). First, each worm explored its surroundings actively. When two worms touched each other, they became entangled (Figure 6B; 0–100 s; Supplementary Video 11). After forming the tangle, the worms continued to actively explore (100~300 sec). The worms moved more actively than the blobs that comprised a large number of worms (Section 2.1.1; Figures 1A, B) in a similar manner as the blackworm (Ozkan-Aydin et al., 2021).

#### 2.3.3. Response to aversive stimuli

We observed the response of worms to the repellent mustard. A worm was placed at the center of 150 mm Petri dish, and a mustard tip was applied near the worm. When mustard was added, worm movement increased (Figure 6C; Supplementary Video 12), compared with those without the aversive stimulus (Section 2.3.1; Supplementary Video 10). It gradually moved away from the stimulus during wiggling. These behaviors are similar to those of a blackworms against aversive water temperatures (Ozkan-Aydin et al., 2021).

### 2.4. Possible mechanism for the collective movement in a confined environment

**Figure 7:**
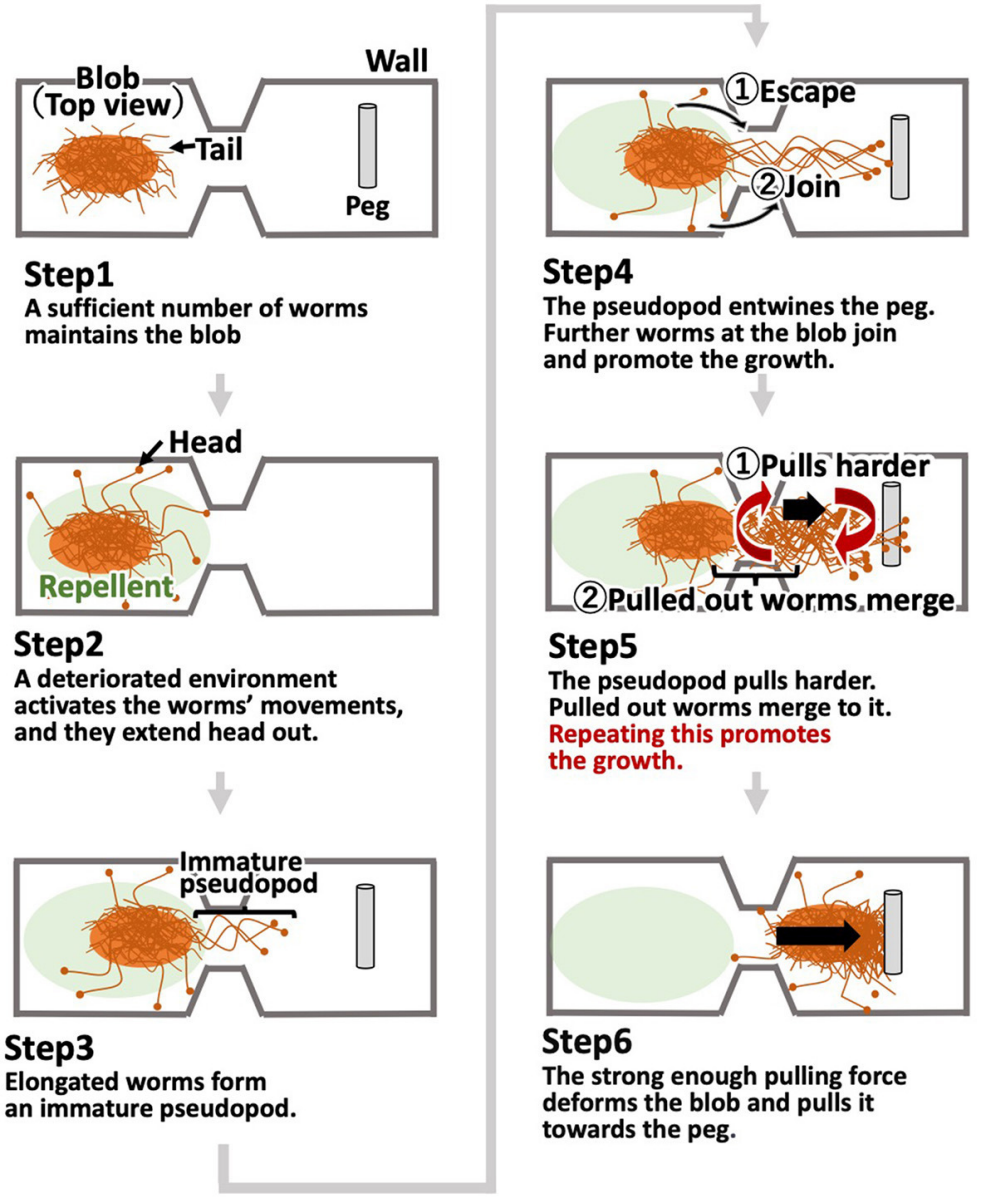
Our working hypothesis for the collective motion of tubificine worms' blobs.

Based on the locomotion findings of one or more worms in Section 2.3, we hypothesized that the motion of a blob in a dumbbell-shaped case with pegs (Figures 3C, D, Section 2.2) occurs by the following steps (Figure 7):

1. When a sufficient number of worms come into contact, they form a blob, in which each worm does not move actively. Worms at the edge of the blob keep their heads inside the blob and their tails outside.
2. Certain substances that work as repellents are produced by worms, which activate their movement and cause some worms to expand their heads out of the blob.
3. The elongated worms out of the blob occasionally come into contact with other worms, leading to the form of an immature pseudopod.
4. This immature pseudopod grows as more worms around it join in. After a time, it entwines pegs. This allows worms at the base of the pseudopod to escape and merge with the pseudopod.
5. As the pseudopod grows in size, the worms in it pull the blob cooperatively, which results in the deformation of the blob. This deformation facilitates the heads of worms in the blob to get out, and they merge with the pseudopod. The pseudopod grows through this positive feedback loop.
6. When the pulling force of the pseudopod becomes strong enough, the entire blob is pulled toward the entwined pegs.

After the aforementioned one-way trip, the blob was assumed to return to its initial room using the same process.

In the following sections, we investigate the validity of this hypothesis using mathematical modeling (Section 3) and simulations (Section 4).

## 3. Mathematical model

We constructed a simple two-dimensional agent-based model. First, we provide an overview of the model (Section 3.1) and then provide detailed explanations (Section 3.2).

### 3.1. Overview

**Figure 8:**
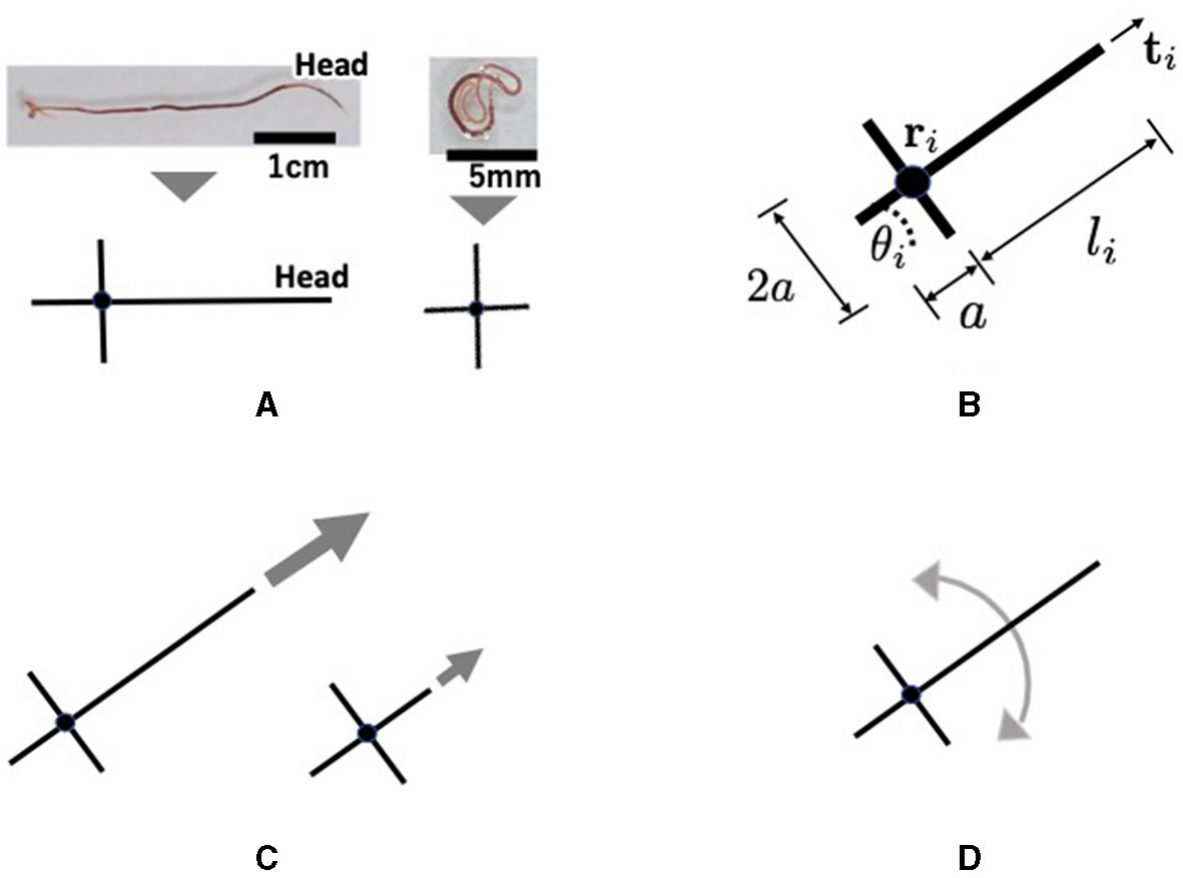
Schematics of the proposed mathematical model: **(A)** Abstract description of a real tubificine worm. expanding (left) and coiling (right) worms are represented by elongated and shortened agents, respectively. The short axis does not change in length, but it promotes entanglement with other agents. **(B)** Definition of variables. *l*_*i*_ changes such like **(A)**. **(C)** Parallel movement. An expanding agent (left) moves faster than a coiling one (right). **(D)** Rotational movement.

Tubicficinae worms propagate waves of expansion and contraction from head to tail to propel them along the body (Figure 6A). It randomly bends its head to perform directional changes and explores its surroundings (Figure 6A). Moreover, real worms exhibit complex three-dimensional entanglement (Figures 1A, B). However, the movements are described in two dimensions in the proposed model for simplicity. Each worm is described by a cross-shaped agent on the *x*−*y* plane (Figure 8A). The agent consists of a longer axis (major axis) and a shorter axis (minor axis); the length of the major axis is adjustable, whereas that of the minor axis is fixed. Specifically, only the length from the intersection point to the head end, denoted by *l*_*i*_, was variable, whereas the length from the intersection point to the other ends was set as a positive constant *a* (Figure 8B). The cross shape represents the area covered by a worm. The expanded and contracted major axes represent the elongated and coiled body postures of the actual worm, respectively (Figure 8A).

Section 2.3 demonstrates that a real worm moves forward by propagating, expanding, and contracting body waves. However, for simplicity in the model, we assumed that an agent moves forward by generating a self-driving force (Figure 8C). It was also assumed that the agent could deform (Figure 8A) and rotate (Figure 8D), based on the finding that a real worm often bends its body and turns (Figure 6). The deformational, translational, and rotational movements of the agent are expressed as follows:


(1) $$\tau {\dot{l}}_{i}={\bar{l}}_{i}-l_{i},$$

(2) $$m{\overset{..}{r}}_{i}+c{\dot{r}}_{i}=F_{i,prop}+\sum_{j\in C_{i}}F_{ij,phys}+F_{i,env},$$

(3) $$\begin{array}{l} \begin{array}{c} {\overset{•}{\theta }}_{i}=W_{i,rand}+W_{i,chem}+\underset{j\in C_{i}}{\sum }W_{ij,phys}+W_{i,env}, \end{array} \end{array}$$

where τ denotes the response time; ${\bar{l}}_{i}$ denotes the target length of *l*_*i*_ which is explained in more detail later (Section 3.2.1); **r**_*i*_ denotes the position of the intersection of the two axes in agent *i*; θ_*i*_ denotes the absolute angle of the major axis (Figure 8B), *m* denotes the mass of the agent, *c* denotes the viscous friction coefficient of the ground, and *C*_*i*_ denotes a set of other agents *j* (≠*i*) in contact with agent *i*. Equation (1) describes the deformation of agent *i*. Equation (2) describes the translational movement of agent *i*. The first term **F**_*i, prop*_ represents the propulsion in the direction of the major axis, the second term **F**_*ij, phys*_ represents the physical interactions between agents, and the third term **F**_*i, env*_ represents the physical interactions between the agent and the environment. Equation (3) describes the rotational movement of the agent. The first term, *W*_*i, rand*_ represents a random rotation that mimics the random turning of a real worm (Figure 6A), the second term *W*_*i, chem*_ represents turning of the head according to aversive stimuli (Figure 6C), the third term *W*_*ij, phys*_ represents physical interaction between agents, and the fourth term *W*_*i, env*_ represents physical interactions between the agent and the walls and between the agent and pegs.

### 3.2. Details of the proposed mathematical model

This section describes the proposed model in detail. First, we explain the deformation and movement of the agent (Section 3.2.1). We then describe the physical interactions between agents (Section 3.2.2). Finally, the physical interaction between the agent and the environment is explained (Section 3.2.3).

#### 3.2.1. Deformation and movements of the agent

The length *l*_*i*_ of part of the major axis changes based on the number of contacting agents *N*_*i*_, considering that real worms tend to assume a coiled posture when in contact with other worms (Figure 1B, Equation 1). ${\bar{l}}_{i}$ denotes the target length of *l*_*i*_ and is expressed by the following equation:


(4) $$\begin{array}{l} {\bar{l}}_{i}=\max\left(l_{a}-l_{b}N_{i},a\right), \end{array}$$

where *l*_*a*_ and *l*_*b*_ are the positive constants. Equation (4) indicates that the length of the major axis *l*_*i*_ becomes shorter as the number of contacting agents *N*_*i*_ increases. When *N*_*i*_ exceeds (*l*_*a*_−*a*)/*l*_*b*_, the lengths of the agent's major and minor axes are equal.

The worms changed their movement depending on the situation (Figures 1B, 6B, C). They became more active as the number of worms in contact with them decreased (Figures 1B, 6B) and as the concentration of aversive stimuli increased (Figure 6C). In this model, we introduce this effect as the activity level *G*_*i*_, which is defined as


(5) $$G_{i}(t)=f\left(\alpha A\left(r_{i}\right)-\beta N_{i}\right)\text{​},$$

where *f*(*x*) denotes the saturation function.


(6) $$f(x)=\{\begin{array}{ll} 1 & (1<x),\\ \frac{1}{2}(1+x) & (-1\leq x\leq 1),\\ 0 & (x<-1). \end{array}$$

α and β are positive constants and *A*(**r**_*i*_) represents the intensity of the aversive stimuli at **r**_*i*_. We hypothesized that a certain repellent substrate would be released from the worms (Section 2.2.1). Thus, we assumed that the aversive stimuli *A*(**r**) were composed of two types: substances released from each agent, denoted by *a*_*s*_(**r**), and mustard added to the behavioral experiment, denoted by *a*_*m*_(**r**). Thus, *A*(**r**) is given by the following equation:


(7) $$A(r)=a_{s}(r)+a_{m}(r).$$

Based on gradual staining of the confined space water (Supplementary Videos 6, 7), the term *a*_*s*_(**r**) is described by the following reaction-diffusion equation:


(8) $${\dot{a}}_{s}(r)=-k_{A_{0}}a_{s}(r)+D_{A}\nabla^{2}a_{s}(r)+k_{A_{1}}\sum_{i}\delta \left(r-r_{i}\right),$$

where *k*_*A*_0__ is the decay coefficient, *k*_*A*_1__ is the release rate of the repellent, and *D*_*A*_ is the diffusion coefficient.

The propulsion of an agent along its major axis **F**_*i, prop*_ is expressed as follows:


(9) $$F_{i,prop}=\eta G_{i}l_{i}t_{i},$$

where η is a positive constant and **t**_*i*_ denotes a unit vector representing the direction of the major axis. Here, *G*_*i*_ and *l*_*i*_ are multiplied on the right-hand side because it is natural to consider that the propulsion force is larger when the agent is more active, and its posture is more elongated.

The random rotation term *W*_*i, rand*_ in Equation (3) is expressed by the following equation:


(10) $$\begin{array}{l} \begin{array}{c} W_{i,rand}=d_{R}\zeta_{i}, \end{array} \end{array}$$

where *d*_*R*_ is a positive constant and ζ_*i*_ is a uniform random number between −1 and 1, which changes at the time interval *T*_*i*_, where *T*_*i*_ is a uniform random number between *t*_*min*_ and *t*_*max*_.

The term *W*_*i, chem*_ functions such that the major axis of agent *i* turns in the direction in which the aversive stimuli decrease (Figure 6C). This is expressed as follows:


(11) $$W_{i,chem}=d_{C}G_{i}\sin\left({\bar{\theta }}_{i}-\theta_{i}\right)\text{​},$$

where *d*_*C*_ is a positive constant, ${\bar{\theta }}_{i}$ denotes the absolute angle of the direction in which aversive stimuli decrease at **r**_*i*_; that is, ${\bar{\theta }}_{i}=\arg\left(-\nabla A{|}_{r=r_{i}}\right)$. Here, *G*_*i*_ is multiplied by the right side of Equation (11), assuming that agents turn strongly when active.

#### 3.2.2. Inter-agent interaction

Real worms interact physically when in contact with one another. This interaction is very complex because the force generated by entanglement (Figures 1A, B) is not a simple frictional force. Here, the interaction force between agents *i* and *j*, **F**_*ij, phys*_, is modeled by a combination of the viscous friction force and the force required to maintain the distance between the agents which denotes the entanglement:


(12) $$F_{ij,phys}=q_{1}({\dot{r}}_{j}-{\dot{r}}_{i})+\rho_{1}\left\{{\left(\frac{\left|r_{ij}\right|}{r_{0}}\right)}^{-\lambda_{1}}-\rho_{2}{\left(\frac{\left|r_{ij}\right|}{r_{0}}\right)}^{-\lambda_{2}}\right\}{\hat{r}}_{ij},$$

where *q*_1_ is a viscosity friction coefficient, ρ_1_, ρ_2_, λ_1_, and λ_2_ are positive constants, **r**_*ij*_ = **r**_*j*_−**r**_*i*_, and $r_{ij}=r_{j}-r_{i},\text{ and }{\hat{r}}_{ij}=r_{ij}/|r_{ij}|$. The first term on the right side of Equation (12) denotes the viscous friction force. The second term works such that the distance between agents *i* and *j* does not become too large or too small.

Next, we explain the physical interaction term for rotational motion *W*_*ij, phys*_. The elongated worms in the pseudopods aligned their bodies when in contact with one another (Figure 3D), whereas worms inside the blob were randomly oriented (Figure 3D). Based on these findings, *W*_*ij, phys*_ can be described as follows:


(13) $$\begin{array}{l} \begin{array}{c} W_{ij,phys}=d_{A}G_{i}\left(l_{i}-a\right)\left(l_{j}-a\right)\sin\left\{2\left(\theta_{j}-\theta_{i}\right)\right\}, \end{array} \end{array}$$

where *d*_*A*_ denotes a positive constant. The term sin{2(θ_*j*_−θ_*i*_)} represents the nematic interaction; that is, the relative angle between agents *i* and *j* approaches either 0 or π so that they are aligned. The terms (*l*_*i*_−*a*) and (*l*_*j*_−*a*) indicate the extent to which agents *i* and *j* are elongated. Therefore, the agents are aligned as they elongate.

#### 3.2.3. Agent-environment interactions

**Figure 9:**
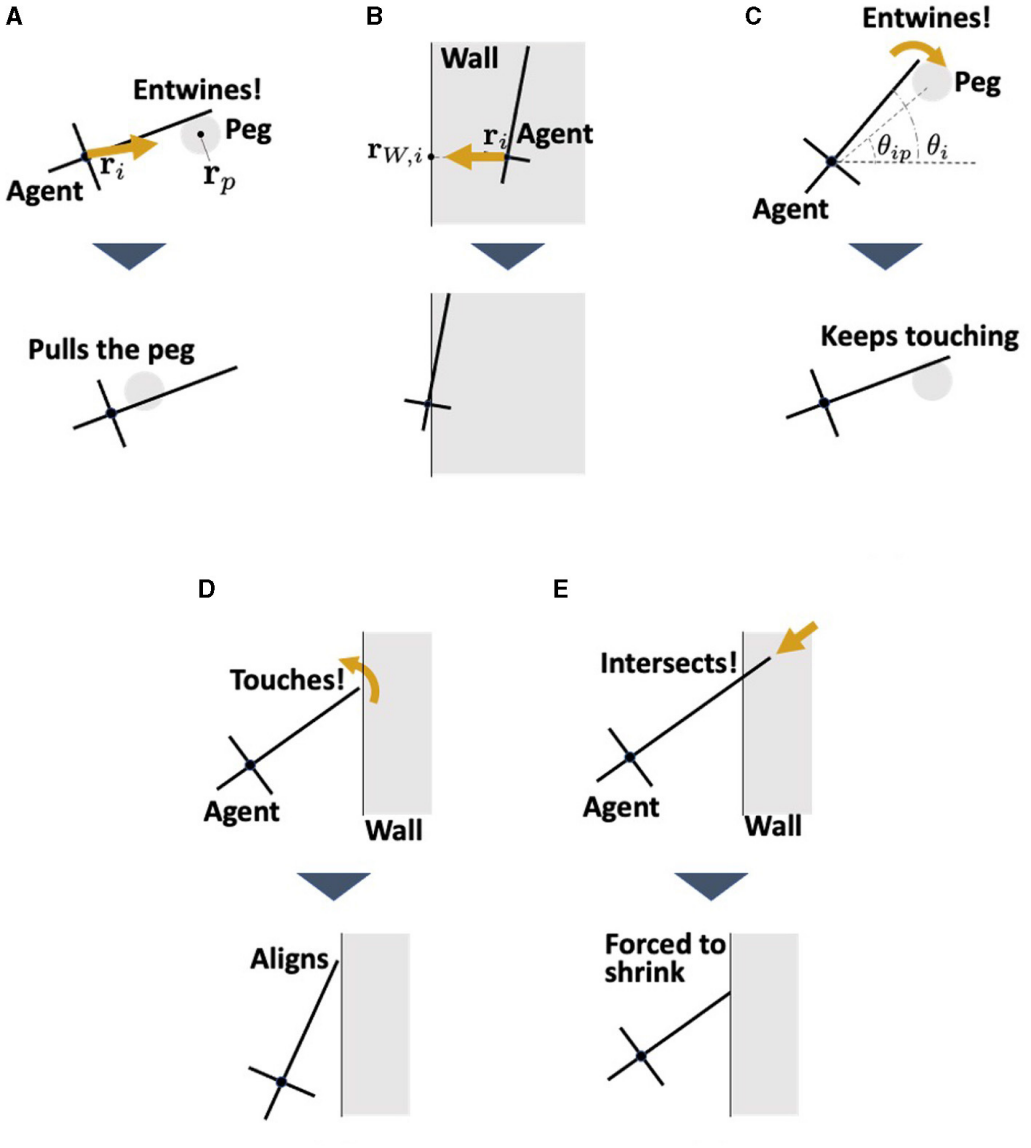
Interaction between the agent and the environments. **(A)** Pulling force against the peg. **(B)** Repulsion force from the wall. This represents the reaction force from the wall when the worm collides with the wall. **(C)** Adjustment the angle θ_*i*_ when the agent touches the peg. **(D)** Alignment the angle θ_*i*_ when the agent touches the wall. This represents the observation that earthworms are propelled along the wall they touch (Figure 3D). **(E)** Shortening of the length *l*_*i*_ when the head end touches the wall. This prevents the agent which touches the wall from penetrating it.

In this model, the agents interact with the pegs and walls (Figure 9). The pegs are modeled as circles of radius *r*_*c*_ which do not move. The physical interaction between the agent and environment for translational motion **F**_*i, env*_ is expressed by the following equation:


(14) $$F_{i,env}=F_{i,peg}+F_{i,wall}.$$

The first term, **F**_*i, peg*_ represents the interaction force between agent *i* and the peg it contacts (**F**_*i, peg*_ = 0 when agent *i* does not contact any peg). This force consists of the viscous friction force and the force from the peg to the agent (Figure 9A); thus, it is described as


(15) $$F_{i,peg}=-q_{2}{\dot{r}}_{i}+\rho_{3}\left(l_{i}-a\right)\left\{{\left(\frac{\left|r_{ip}\right|}{r_{0}}\right)}^{-\lambda_{1}}-\rho_{2}{\left(\frac{\left|r_{ip}\right|}{r_{0}}\right)}^{-\lambda_{2}}\right\}{\hat{r}}_{ip},$$

where *q*_2_ is a viscous friction coefficient, ρ_3_ is a positive constant, **r**_*ip*_ = **r**_*p*_−**r**_*i*_, and $r_{ip}=r_{p}-r_{i},\text{ and }{\hat{r}}_{ip}=r_{ip}/|r_{ip}|$ with **r**_*p*_ being the position of the center of peg *p*. The second term in Equation (15) represents the dragging force from agent *i* to the peg (Figures 3C, D). The term *l*_*i*_−*a* in the second term indicates that a stronger force is generated as agent *i* elongates. This was based on the assumption that an elongating agent entwining a peg produces a strong drag force (Ozkan-Aydin et al., 2021).

The second term **F**_*i, wall*_ in Equation (14) represents the repulsion force from the wall with which agent *i* contacts (**F**_*i, wall*_ = 0 when agent *i* does not contact any wall). This is simply described as a force proportional to the penetration length to the wall (Figure 9B), that is,


(16) $$F_{i,wall}=k_{W}(r_{W,i}-r_{i}),$$

where *k*_*W*_ is a positive constant and **r**_*W*.*i*_ denotes the nearest point of the wall surface from **r**_*i*_.

The physical interactions with the environment for the rotational motion of agent *i*, denoted *W*_*i, env*_, are described as


(17) $$\begin{array}{l} \begin{array}{c} W_{i,env}=W_{i,peg}+W_{i,wall}, \end{array} \end{array}$$

where *W*_*i, peg*_ represents the interaction with the peg which agent *i* contacts, expressed as


(18) $$\begin{array}{l} \begin{array}{c} W_{i,peg}=d_{P}\text{sin}2\left(\theta_{ip}-\theta_{i}\right), \end{array} \end{array}$$

where *d*_*P*_ is a positive constant and θ_*ip*_ denotes the angle of the center of the contacting peg *p* viewed from **r**_*i*_; that is, θ_*ip*_ = arg(**r**_*p*_−**r**_*i*_). This term operates such that agent *i* is aligned in the direction of the contact peg (Figure 9C). When agent *i* does not contact any of the pegs, *W*_*i, peg*_ = 0.

The second term of Equation (17), *W*_*i, wall*_, represents the interaction with a wall which agent *i* contacts, which is expressed by the following equation:


(19) $$\begin{array}{l} \begin{array}{c} W_{i,wall}=d_{W}\sin2\left(\theta_{u}-\theta_{i}\right), \end{array} \end{array}$$

where *d*_*W*_ is a positive constant and θ_*u*_ is the absolute angle of the wall that agent *i* contacts. This term operates such that the agent *i* aligns with the contact wall (Figure 9D), based on the finding that worms move along walls (Figure 3D). When agent *i* does not make contact with any walls, *W*_*i, wall*_ = 0.

In addition, when the head end of agent *i* touches the wall, *l*_*i*_ is forcibly changed so that it does not penetrate the wall (Figure 9E).

## 4. Simulation

To test the validity of our model, we simulated the collective behaviors observed in biological experiments (Sections 2.1 and 2.2). The simulation was coded as C++. The time evolutions of **r**_*i*_ and θ_*i*_ were solved using the two-stage Runge-Kutta method, and that of *l*_*i*_ was solved using the Euler method. The time interval d*t* was set to 0.2 s. The number of total time steps in each trial depends on the experiment (10 × 10^3^, 100 × 10^3^, and 400 × 10^3^ steps in the experiments described in Sections 4.1.1, 4.1.2, and 4.2, respectively). The velocity ${\dot{r}}_{i}$ was initially set to 0. The initial value of *l*_*i*_ was set to *a*. *a*_*s*_(**r**) was initially set to 0 throughout the environment. The Neumann boundary condition was used to solve Equation (8). The mass *m* and length of the fully elongating agent *l*_*a*_ were determined based on those of real worms. In contrast, the other parameter values were hand-tuned because they could not be estimated from biological findings (Table 1).

**Table 1:** Parameters employed in the simulations.

**Parameter**		**[Unit]**	**Value**
*m*	Mass of an agent	[kg]	1.0 × 10^−3^
*c*	Ground friction coefficient	[kg/s]	0.10
*a*	Length of the agent's minor axis	[m]	2.5 × 10^−3^
τ	Time constant for changing axis length	[s]	100.0
*l*_*a*_	Positive constant for the axis length	[m]	0.05250
*l*_*b*_	Positive constant for the axis length	[m]	2.5 × 10^−3^
α	Parameter related to activity level (Equation 5)	[−]	1.2
β	Parameter related to activity level (Equation 5)	[−]	0.05
η	Parameter related to the propulsive force of the agent's translational motion	[s^2^/kg]	5.0 × 10^3^
*d*_*R*_	Parameter related to the maximum torque of the agent's random bending	[1/s]	0.0124
*t*_*min*_	Minimum time interval for updating torque	[s]	2.0
*t*_*max*_	Maximum time interval for updating torque	[s]	20.0
*d*_*C*_	Parameter related to the aligning torque Toward the direction with fewer aversive stimuli	[1/*s*]	7.5 × 10^−3^
*q*_1_	Friction coefficient between agents	[kg/s]	1.88 × 10^−3^
ρ_1_	Parameter related to the attractive force toward another agent	[kg·m/s^2^]	0.165 × 10^−6^
ρ_2_	Parameter related to the repulsive force against another agent	[−]	0.19
ρ_3_	Parameter related to the pulling force against the peg	[kg/s^2^]	11.6 × 10^−6^
λ_1_	Positive constant related to entangling between agents	[−]	1.0
λ_2_	Positive constant related to entangling between agents	[−]	2.0
*r*_0_	Positive constant related to entangling between agents	[m]	0.01
*d*_*A*_	Aligning torque toward another agent	[1/m^2^s]	53.3
*q*_2_	Viscous friction coefficient against the peg	[kg/s]	0.125 × 10^−3^
*d*_*P*_	Parameter related to the aligning torque toward the peg	[1/s]	0.333 × 10^−3^
*d*_*W*_	Parameter related to the aligning torque toward the wall	[1/s]	1.0 × 10^−3^
*k*_*W*_	Spring coefficient of the wall	[1/s]	3.0 × 10^−3^
*D*	Parameter related to the mustard distribution	[−]	0.61
*E*	Parameter related to controlling the mustard distribution	[−]	0.125
*k*_*A*_0__	Decay coefficient of aversive stimuli from the agent	[−]	5.71 × 10^−3^
*k*_*A*_1__	Emission rate of aversive stimuli from the agent	[−]	1.0 × 10^−3^
*D*_*A*_	Diffusion coefficient of aversive stimuli from the agent	[−]	0.07

### 4.1. Simulation results in a flat environment

#### 4.1.1. Blob formation

To imitate the blob-formation experiment in a box (Figure 1A), we set a square-shaped wall of length 0.183 m on one side. As the initial condition, 1, 000 agents were randomly deployed throughout the field, and their angles were randomly distributed. No external aversive stimulus was added in this experiment; that is, *a*_*m*_(**r**) = 0.

**Figure 10:**
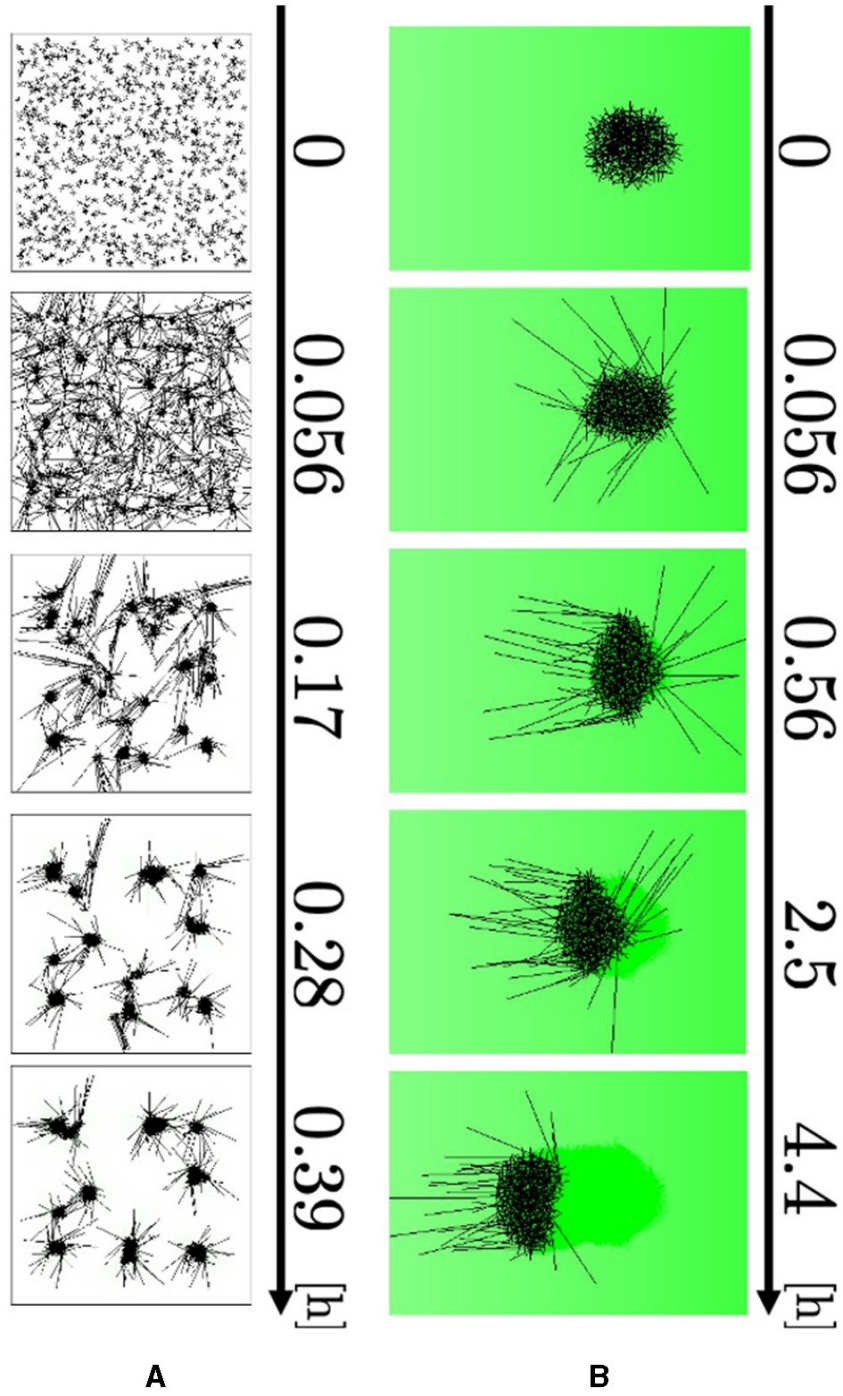
Simulation results. **(A)** Blob formation. **(B)** Collective escape. Dark green indicates the intensity of aversive stimuli *A*(**r**).

The agents elongated their major axes and attracted the neighboring axes to form small blobs (Figure 10A, 0–0.056 h; Supplementary Video 13). The blobs grew by absorbing the surrounding agents and attracted one another to form larger blobs (0.056–0.39 h). Therefore, the number of blobs decreased. These results are consistent with biological findings (Figure 1A).

#### 4.1.2. Collective escape from aversive stimulus

We simulated collective escape from the repellent mustard (Figure 1C). Simulations were performed using open boundaries. Two hundred and fifty agents, whose angles were randomly set, were initially located within a circle of radius 0.0125 m centered on the origin, such that they formed a blob at the beginning. The concentration gradient of real mustard was simulated by setting the stimulus term *a*_*m*_(**r**) to


(20) $$a_{m}(r)=D\text{exp}(Ex),$$

where *D* and *E* are the positive constants.

The agents at the edge of the blob begin to expand their bodies and move their heads out of the blob because they have a small number of contacting agents *N*_*i*_ (Figure 10B, 0–0.056 h; Supplementary Video 14). They turned their heads in the negative direction of the *x* axis, to which the aversive stimulus decays (0.056–0.56 h). After that, they dragged the other agents so that the blob moved straight toward the direction in which the stimulus decays (0.56–4.4 h). Therefore, the simulation qualitatively reproduced responses to an aversive stimulus.

### 4.2. Collective self-transport in a confined environment

We investigated whether our model could reproduce the behavior described in Section 2.2. Specifically, the simulations were performed using boundary conditions that imitated the cases used in the experiments (Figure 2): oval-shaped boundary (Major axis; 0.05 m, minor axis: 0.025 m), and a dumbbell-shaped boundary (Major axis; 0.05 m, minor axis: 0.025 m) consisting of two regular octagons connected by a narrow passage. Simulations were performed by placing several pegs in the rooms of the dumbbell-shaped case. Two hundred and fifty agents with randomly set angles were placed on the left side within a circle of radius 0.0125 m to form the blob. No aversive stimuli were added in this experiment; that is, *a*_*m*_(**r**) = 0. We performed 50 trials on each simulation.

**Figure 11:**
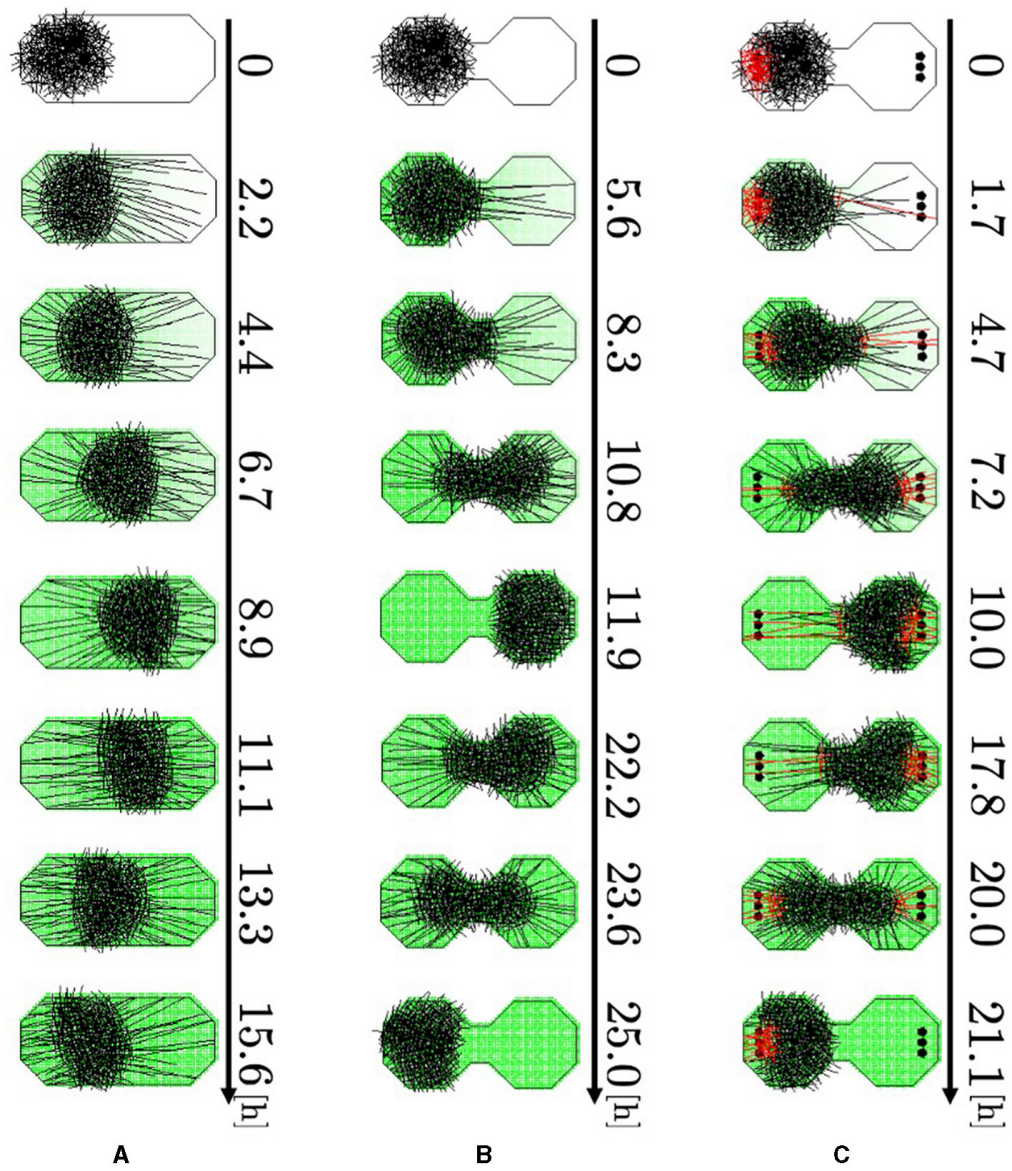
Simulation results. **(A)** Oval-shaped case. **(B)** Dumbbell-shaped case without pegs. **(C)** Dumbbell-shaped case with pegs. Red agents are touching pegs. Dark green indicates the intensity of the aversive stimuli *A*(**r**).

Representative snapshots of the oval-shaped case show that the blob moved straight toward the other side (Figure 11A, 0–8.9 h; Supplementary Video 15a). Subsequently, it returned to its initial position (8.9–15.6 h). In the dumbbell-shaped boundary without pegs, the blob attempted to move toward the opposite room but was unable to pass through the narrow path for some time (Figure 11B, 0–5.6 h; Supplementary Video 15b). Subsequently, the blob deformed to enter the path, and the entire blob finally reached another room (5.6–11.9 h). After the blob reached the room, it returned to the initial room using a similar process (11.9–25.0 h). In the dumbbell-shaped boundary with pegs (Figure 11C; Supplementary Video 15c), some agents got the heads out of the blob, and then they entwined pegs at the destination (0–1.7 h). They exerted dragging forces, and consequently, the blob deformed and moved to another room (1.7–10.0 h). Subsequently, the blob returned to its initial room using a similar process (10.0–21.1 h).

**Figure 12:**
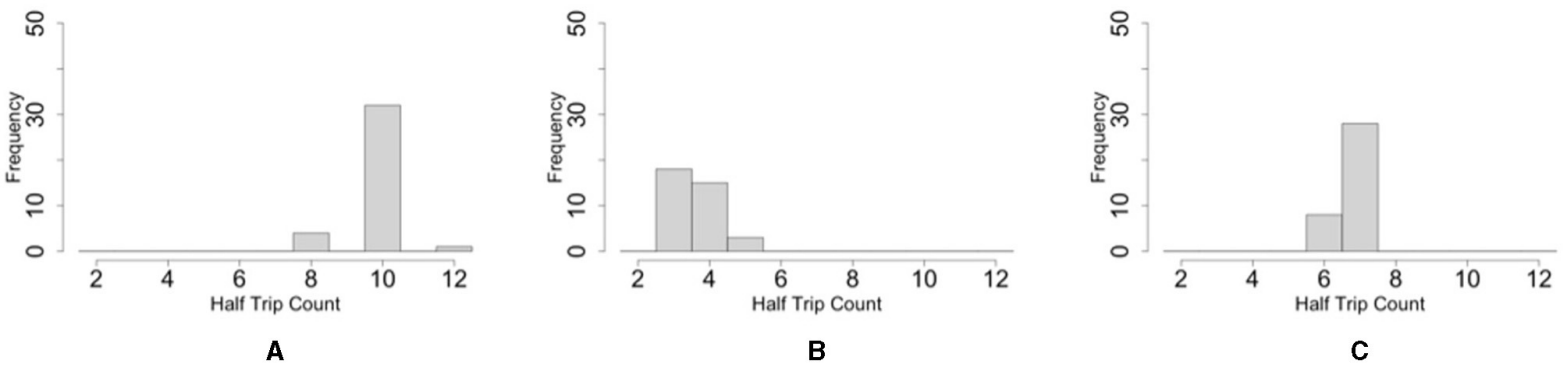
Histograms of the number of one-way trips of the blob on the simulations. **(A)** Oval-shaped case. **(B)** Dumbbell-shaped case without pegs. **(C)** Dumbbell-shaped case with pegs.

The histogram shows that the number of one-way trips of the blobs under the dumbbell-shaped boundary without pegs was significantly lower than that under the oval boundary (Figures 12A, B; *p* < 0.001, Mann–Whitney *U*-test). Furthermore, the number of one-way trips of the blobs under the condition of a dumbbell-shaped boundary with pegs was significantly higher than without pegs (Figures 12B, C; *p* < 0.001, Mann–Whitney *U*-test). These results are consistent with those of biological experiments (Section 2.2).

## 5. Discussion

We have studied the mechanism for adaptive locomotion of worm blobs. Although previous studies on worm blobs (Nguyen et al., 2021; Ozkan-Aydin et al., 2021) focused on the mechanism of thermotaxis, their focus was limited to locomotion in a flat environment. In contrast, we aimed to clarify how a worm blob adaptively moves in real-world environments with confined spaces and environments by exploiting physical connection-based interactions among the constituting worms. Thus, we observed the movement of tubificine worm swarms in a confined space with heterogeneity and several other environments (Figures 1, 3). We found a novel behavior in which they used pegs that the worms could entwine. These results suggest that the worm blobs maintained their hemispherical shape in the open arena. However, in a confined channel with several pegs, the blob could deform flexibly and move effectively using the pegs. Specifically, a pseudopod is generated from a blob and entwines pegs, which leads to the effective drag of the entire blob. Based on these findings, we constructed a simple 2D agent-based model in which the flexible body of a real worm is represented by a cross-shaped agent that can deform, rotate, and translate (Figure 8). Through simulations, we successfully reproduced the observed behavior (Figures 1, 3) by using common parameters (Figures 10, 11).

However, the biological advantages of these behaviors in worms remain unclear. We believe that maintaining the hemispherical shape of the blob can protect worms from environmental degradation and predators like as the blackworm blob (Ozkan-Aydin et al., 2021). This shape allows the entire blob to move in a stable direction (Figure 1A), whereas worm pieces move around in a disorderly manner (Figure 6B). However, maintaining a hemispherical shape when passing through confined spaces in natural environments is impractical (Figure 3B). In a confined environment, the worm blob likely overcomes this disadvantage by using “exploration and exploitation (Del Ser et al., 2019)” through pseudopod formation (Figures 3C, D). This strategy allows the blob to survive in harsh environments.

There are similarities and differences between tubificine worms and other long, soft-structured organisms. For example, an amoeba moves by expanding its pseudopodia and controlling the alignment and distribution of its fibers inside the body (Swanson and Taylor, 1982). *C. elegans* has some similar features of nematic interaction to form bundles in aligned orientation (Sugi et al., 2019). Moreover, *C. elegans* tends to aggregate as population density increases (Ding et al., 2019). By contrast, *C. elegans* cannot be tightly entangled because of its small aspect ratio. Such comparisons among different organisms are important for a systematic understanding of the principles inherent in physical-connection-based swarms and require further validation. In particular, the behavior of other organisms in confined environments remains largely unclear; thus, the applicability of our findings to other organisms needs to be investigated in the future.

From an engineering perspective, the proposed mechanism for tubificine worms could lead to the development of a new type of swarm robotic system. In contrast to previous swarm robotic works that used rigid robots (Brambilla et al., 2013; Hamann, 2018; Schranz et al., 2020), tubificine worms can change their behavior in a variety of ways through the deformation and entanglement of their soft bodies and can adapt flexibly to unstructured environments. Therefore, we expect that a swarm of soft and elongated robots that implement the tubificine worm mechanism will function in complex natural environments where conventional swarm robot systems made of rigid robots cannot work.

In experiments conducted in a confined environment (Figure 3), we assumed that the blobs moved back and forth, driven by the self-induced chemical stimulus. Another possible mechanism of blob motion is that the edge worms anchor themselves to the surrounding walls and pull the blobs forward. However, we believe that chemical stimuli work predominantly to trigger the motion of blobs for the following reasons. First, if the edge worms anchored themselves to the surrounding walls, the blobs should deform along the walls; however, in the oval-shaped experiment (Supplementary Video 4), the blob moved while maintaining its hemispherical shape. Second, based on the finding that annelids excrete toxic nitrogenous waste (Cohen and Lewis, 1949; Thiel et al., 2017) and that several animals avoid high concentrations of ammonia (Grewal et al., 1993; Richardson et al., 2001; Clark et al., 2023), it is not surprising that tubificine worms release nitrogenous compounds that self-induce avoidance behaviors. However, further investigation is required, and the identification of self-induced aversive substances is a topic for future research.

There remain several other future works from both biological and mathematical perspectives. First, although we recapitulated their behavior in confined cases, it remains challenging to explain them in more complex environments. Further behavioral experiments in complex environments and the elaboration of a mathematical model on this basis are needed to address this issue. Second, although the blob moved back and forth with irregular periods in the behavioral experiments described in Section 2.2 (Figures 3, 4), it is difficult to reproduce the irregularity in our model. This discrepancy may have originated from the heterogeneity of worm properties, such as locomotor abilities and responses to stimuli. When heterogeneity exists, the timing of the initiation of the blob's motion may depend on the properties of the worm. However, this requires further clarification in future studies. Third, unlike real worms, the agent in our model did not have bending softness. Therefore, some of the behaviors exhibited by real worms may be difficult to reproduce. For example, a real worm can move by aligning its body with the heterogeneity of its environment. However, it is difficult to reproduce this behavior in our model. Extensions to our mathematical model are required to address these issues.

## Data availability statement

The original contributions presented in the study are included in the article/Supplementary material, further inquiries can be directed to the corresponding author.

## Author contributions

TM and TK conceived the work. TM, TK, and RK proposed the mathematical model and all authors refined it. TM and DW conducted the biological experiments. TM, DW, TK, and AI analyzed the results. TM wrote the code and performed mathematical simulations. All authors gave final approval for publication and agreed to be held accountable for the work performed therein.
