# Supplementary material for: Elongating, entwining, and dragging: mechanism for adaptive locomotion of tubificine worm blobs in a confined environment
Source: Front Neurorobot. 2023 Aug 29;17:1207374. doi: 10.3389/fnbot.2023.1207374 (PMC10495593; doi:10.3389/fnbot.2023.1207374)
Supplement: Supplementary file 1 [file Data_Sheet_1.pdf]

Title of Dataset
Supplementary videos for the paper ``Elongating, entwining, and dragging: mechanism for adaptive locomotion of tubificine worm blobs in a confined environment”

Description of the data and file structure

Supplementary Video 1:
Blob formation (Fig. 1A). 5x speed.

Supplementary Video 2:
Stationary worm blob (Fig. 1B). 10x speed.

Supplementary Video 3:
Collective escape from a repellent (mustard) (Fig. 1C). 100x speed.

Supplementary Video 4:
Collective locomotion in an oval-shaped case (Fig. 3A). 3000x speed.

Supplementary Video 5:
Collective locomotion in a dumbbell-shaped case without pegs (Fig. 3B). 3000x speed.

Supplementary Video 6:
Collective locomotion in oval-shaped cases for statistics (Fig. 5A). 15000x speed.

Supplementary Video 7:
Collective locomotion in dumbbell-shaped cases for statistics (Fig. 5B, C). Pegs are applied in the upper three rows, but not in the lower three rows. 15000x speed.

Supplementary Video 8:
Collective locomotion in a dumbbell-shaped case with pegs (Fig. 3C). 3000x speed.

Supplementary Video 9:
Pseudopod formation in a dumbbell-shaped case (Fig. 3D). 2.5x speed.

Supplementary Video 10:
Movement of a single worm (Fig. 6A). 10x

speed.

Supplementary Video 11:
Entanglement of two worms (Fig. 6B). 50x speed.

Supplementary Video 12:
Response of a single worm to a repellent (mustard) (Fig. 6C). 10x speed.

Supplementary Video 13:
Simulation of the blob formation (Fig. 10A).

Supplementary Video 14:
Simulation of the collective escape from a repellent (Fig. 10B).

Supplementary Video 15:
Simulation of the collective locomotion in a confined environment. (a) Oval-shaped boundary (Fig. 11A). (b) Dumbbell-shaped boundary without pegs (Fig. 11B). (c) Dumbbell-shaped boundary with pegs (Fig. 11C).
